# In vivo correlation of serotonin transporter and 1B receptor availability in the human brain: a PET study

**DOI:** 10.1038/s41386-022-01369-3

**Published:** 2022-07-11

**Authors:** Jonas E. Svensson, Mikael Tiger, Pontus Plavén-Sigray, Christer Halldin, Martin Schain, Johan Lundberg

**Affiliations:** 1 Centre for Psychiatry Research, Department of Clinical Neuroscience, Karolinska Institutet & Stockholm Health Care Services, Region Stockholm, Karolinska University Hospital, SE-171 76 Stockholm, Sweden; 2 Neurobiology Research Unit, Copenhagen University Hospital, Copenhagen, Denmark; 3 Antaros Medical AB, Bioventure Hub, Mölndal, Sweden

**Keywords:** Brain, Neurotransmitters

## Abstract

Synaptic serotonin levels in the brain are regulated by active transport into the bouton by the serotonin transporter, and by autoreceptors, such as the inhibitory serotonin (5-HT) 1B receptor which, when activated, decreases serotonin release. Animal studies have shown a regulatory link between the two proteins. Evidence of such coupling could translate to an untapped therapeutic potential in augmenting the effect of selective serotonin reuptake inhibitors through pharmacological modulation of 5-HT_1B_ receptors. Here we will for the first time in vivo examine the relationship between 5-HT_1B_ receptors and serotonin transporters in the living human brain. Seventeen healthy individuals were examined with PET twice, using the radioligands [11C]AZ10419369 and [^11^C]MADAM for quantification of the 5-HT_1B_ receptor and the 5-HT transporter, respectively. The binding potential was calculated for a set of brain regions, and the correlations between the binding estimates of the two radioligands were studied. [^11^C]AZ10419369 and [^11^C]MADAM binding was positively correlated in all examined brain regions. In most cortical regions the correlation was strong, e.g., frontal cortex, r(15) = 0.64, *p* = 0.01 and parietal cortex, r(15) = 0.8, *p* = 0.0002 while in most subcortical regions, negligible correlations was observed. Though the correlation estimates in cortex should be interpreted with caution due to poor signal to noise ratio of [^11^C]MADAM binding in these regions, it suggests a link between two key proteins involved in the regulation of synaptic serotonin levels. Our results indicate a need for further studies to address the functional importance of 5-HT_1B_ receptors in treatment with drugs that inhibit serotonin reuptake.

## Introduction

Major depressive disorder (MDD) is ranked by WHO as the third leading cause of years lost to disability, followed by migraine (position six) and anxiety disorders (position eight). Together they account for 13% of years lost to disability globally [1]. The pathophysiology of these brain disorders is largely unknown but they do share a commonality in that the serotonin (5-HT) system is implicated in all three [2–4]. The first choice of pharmacological treatment for MDD and most anxiety disorders are selective serotonin reuptake inhibitors (SSRIs) [5, 6], blocking the 5-HT transporter (5-HTT) [7]. The same class of drugs can be effective when treating migraine [8], alongside triptanes. In contrast to SSRIs, triptanes act as agonists at the 5-HT 1B receptor (5-HT_1B_) and are recommended as acute treatment for migraine headaches [9]. Modulation of the 5-HT_1B_ receptor has also been suggested to be efficacious in psychiatric conditions [10, 11]. In Sweden around 10% of all citizens filled a prescription of an SSRI 2019 [12]. There is a large unmet need in improving the efficacy and speed of therapeutic onset of SSRIs. Augmentation with 5-HT_1B_ modulating drugs could be effective in this regard [13]. To effectively design such treatments schemes the in vivo relationship between the two proteins should be better mapped out.

The serotonin transporter and the two G-protein coupled, inhibitory, 5-HT receptors 1A and 1B are central to the regulation of serotonergic activity in the brain [14]. 5-HTT is exclusively located pre-synaptically and reduces synaptic 5-HT levels through transport back into serotonergic neurons [14]. 5-HT 1A and B receptors act both as heteroreceptors in projection areas, and as autoreceptors. 5-HT_1A_ autoreceptors are predominantly located in the raphe nuclei, while 1B receptors are located presynaptically also in projection areas. Upon binding to a 5-HT_1B_ autoreceptor, 5-HT inhibits formation of cAMP and downstream cellular responses, thus decreasing release of 5-HT to the synapse [11]. Interestingly, a direct link between 5-HT_1B_ autoreceptors and 5-HTT have been suggested, with increased serotonin reuptake upon 5-HT_1B_ receptor activation in synaptosomes, as well as decreased 5-HTT activity after 5-HT_1B_ receptor antagonist administration [15, 16]. Vice versa, exposure to SSRIs have been shown to decrease 5-HT_1B_ receptor mRNA and 5-HT_1B_ receptor availability in the raphe nuclei in rats and non-human primates [10, 17], and increase 5-HT_1B_ receptor availability in cortical areas in humans [18]. While 5-HT_1A_ receptor modulation have been extensively explored with regards to psychiatric disorders in general and, more specifically, has been suggested to augment the effect of SSRI treatment in depression [19], relatively little focus has been directed towards the 5-HT_1B_ receptor in this respect. Given that both 5-HTT and 5-HT_1__B_ are key proteins in the regulation of 5-HT signal transduction, there could well exist an untapped potential in concomitant SSRI treatment and pharmacological modulation of 5-HT_1B_ receptors.

Positron emission tomography (PET) studies have shown a high correlation between brain regions for the in vivo concentration of most examined proteins related to the serotonin system [20]. This means that if an individual has a high concentration (relative to other individuals) of 5-HT_1B_ in e.g., occipital cortex the same individual is likely to have a high concentration of 5-HT_1B_ also in other brain regions. However less is known of the covariance *between proteins*, e.g., if an individual has high levels of 5-HTT in one brain region, does this predict high levels of 5-HT_1B_ in the same region? Knowledge of the interplay between the proteins regulating 5-HT neurotransmission could generate novel treatment strategies. To this end, several published PET studies have explored the correlation between 5-HTT and 5-HT_1__A_ receptors in healthy subjects indicating brain region specific correlations, with some variability between studies [21, 22]. To our knowledge there exists no study reporting on the correlation between 5-HTT and 5-HT_1B_ receptors in vivo. With the development of selective 5-HT_1B_ radioligands such as [^11^C]AZ10419369 [23], 5-HT_1B_ receptor binding can now be examined in relation to 5-HTT in vivo.

In this study we examined 17 healthy controls with PET and two radioligands during the same day: [^11^C]MADAM and [^11^C]AZ10419369, to quantify the density of 5-HTT and 5-HT_1B_ respectively. This aim of the study was to examine correlations between the two proteins in relevant brain regions.

## Materials and methods

### Subjects

Seventeen volunteers (13 females; 4 males) were recruited. Recruitment was done by advertisement in local newspapers, followed by a physical visit where subjects were screened by a psychiatrist or a resident physician supervised by a psychiatrist, with: (1) the Mini- International Neuropsychiatric Interview (M.I.N.I.) [24]; (2) a physical examination, including a neurological examination; (3) blood tests (complete blood count, aspartate amino transferase, alanine amino transferase, gamma-glutamyl transferase, sodium, potassium, creatinine, calcium, albumin, thyroid stimulating hormone, glucose) and urine tests (drug screen; pregnancy test when indicated); and (4) MRI of the brain. All subjects had no history of disease involving the central nervous system, including current or previous psychiatric disorders, and were aged 23–75 years (47 ± 14, mean ± SD). The study was approved by the Research Ethics Committee in Stockholm, Sweden, and the Radiation Safety Committee at Karolinska University Hospital, Stockholm. All subjects gave verbal and written informed consent before participation. Part of the data have previously served as healthy control data for a longitudinal 5-HTT PET study on depression [25].

### Radiochemistry

Subjects were examined with PET and [^11^C]MADAM (for quantification of 5-HTT) and [^11^C]AZ10419369 (for quantification of 5-HT_1B_ receptors) on the same day and in a random order. For details on radioligand preparation see [26] for [^11^C]MADAM and [23] for [^11^C]AZ10419369. The injected radioactivity for [^11^C]MADAM was 408 ± 85 MBq, the specific radioactivity was 231 ± 134 GBq/μmol, and injected mass was 0.93 ± 1.28 μg. For [^11^C]AZ10419369 the injected radioactivity was 382 ± 63 MBq, the specific radioactivity was 330 ± 183 GBq/μmol, and injected mass was 0.82 ± 0.78 μg.

### MRI and PET experimental procedure

T1-weighted MRI images were acquired using a 3 T GE Signa system (GE Medical Systems, USA). A high-resolution research tomograph (Siemens Molecular Imaging, USA) with a maximum spatial resolution of ~2 mm full-width-half-maximum [27] was used for all PET examinations. Transmission scans were performed prior to each PET measurement to correct for signal attenuation.

In each PET-experiment a saline solution containing [^11^C]MADAM was injected into a antecubital vein as a bolus (<10 s). The cannula was then flushed with 10 mL saline. For both radioligands emission data were acquired continuously for 93 minutes, and subsequently binned into consecutive time frames. For [^11^C]MADAM 38 frames with the following definitions: nine 10 s, two 15 s, three 20 s, four 30 s, four 1 min, four 3 min and twelve 6 min frames and for [^11^C]AZ10419369 37 frames: eight 10 s, five 20 s, four 30 s, four 1 min, four 3 min and twelve 6 min frames.

### Regions of interest

FreeSurfer (version 6.0, http://surfer.nmr.mgh.harvard.edu/) [28] was used to delineate brain regions on the T1-weighted MRIs of all subjects. We chose to quantify the binding in brain regions where both radioligands have a moderate to high binding: amygdala, anterior cingulate gyrus (ACC), posterior cingulate gyrus (PCC), caudate, hippocampus, insular cortex, putamen, and thalamus, but also in cortical regions of interest for both neurological and psychiatric diseases where the [^11^C]MADAM binding is comparatively low: occipital, parietal, frontal and temporal cortex (see Supplementart Fig. S1 for a visualization of the included brain regions). Both radioligands have high binding in pallidum, however this region was not included since equilibrium is not reached during the time of the PET-experiment for either radioligand [29].

Delineation of raphe was performed using an automatic process, described previously [25], where information from FreeSurfer brainstem [30] and the time-weighted summated [^11^C]MADAM image is used to generate a median raphe mask containing 65 voxels and a dorsal raphe mask of 116 voxels (see Supplementary Fig. S2 for a visualization of the raphe masks).

### Image preprocessing and quantification

Dynamic PET images were corrected for head motion using a between-frame-correction algorithm implemented in SPM12 (Wellcome Department of Cognitive Neurology, University College, London, UK). Frames were realigned to the first six-minute frame (21–27 min of the scan for both radioligands). For each individual, the T1-weighted MR-image was co-registered to time-weighted summated PET-images yielding one co-registration matrix per radioligand. The co-registration matrix was then used to project regions of interest (ROIs) on the realigned dynamic PET-image to derive regional time-activity curves.

From the time-activity curves binding potential (*BP*_ND_) was calculated for each ROI using the non-invasive Logan plot fitted with multilinear regression [31]. For [^11^C]AZ10419369, t* was set to 33 min, corresponding to 10 frames. For [^11^C]MADAM, a t* of 45 min (corresponding to 8 frames) was used in order to account for the high density of 5-HTT in the raphe nuclei [32]. The reference region efflux rate constant (k2’) was derived using the simplified reference tissue model [33]; for [^11^C]MADAM the k2’ value from putamen was used [34] and for [^11^C]AZ10419369 average k2’ in frontal- and occipital cortex, caudatus, insula and putamen was used. Cerebellar gray matter was defined as described previously [35] and used as reference region for both radioligands [29, 36].

To extract *BP*_ND_ for the raphe nuclei masks, parametric images were generated using the 3D stationary wavelet aided parametric imaging procedure, where the non-invasive Logan plot, fitted with multilinear regression, is applied on time activity curves from individual voxels [37]. This method has been shown to effectively reduce noise in small brain regions [38]. For each individual, the dorsal and median raphe masks were applied both to the [^11^C]MADAM and [^11^C]AZ10419369 parametric-images to obtain the corresponding binding estimates in these small brainstem regions.

For visualizations, the parametric images were registered to MNI-space [39] and averaged across individuals for both radioligands.

### Statistics

We had no a priori hypothesis of the direction of a putative correlation, or in which brain regions an association would be most likely. Based on this, we performed an exploratory analysis of the data. Thus, we did not apply the conventional null hypothesis significance testing, where an alpha is set for error control, but rather we applied a fisherian interpretation of the calculated *p* values [40], where a low p-value is interpreted as indirect inductive evidence against the null hypothesis (here defined as no association between the radioligands); the smaller the p-value, the more unlikely is the occurrence of these data in the event that the null hypothesis is true (i.e., the more probable explanation is that the null-hypothesis is false and should be rejected). Since no claims regarding type 1 error rate was made, no correction for multiple comparisons was performed. It is possible also under a strict fisherian interpretation to set a threshold for “significance”. However, since this terminology is intimately connected to type 1 error control under null hypothesis significance testing framework, and might lead to confusion, we did not apply any *p* value threshold, nor did we use the term “significance” here. To describe the strength of the association between variables, we used the following heuristic: a correlation estimate between 0–0.19 was described as negligible, 0.2–0.39 as weak, 0.40–0.59 as moderate, 0.6–0.79 as strong and 0.8–1 as very strong [41].

Both 5-HTT and 5-HT_1B_ have been shown to decrease with age [18, 42], a positive correlation between the two radioligands can therefore be expected even with no true underlying association between the examined proteins, or a true association might be masked by noise added by an age effect. Partial Pearson correlation coefficients were therefore calculated, corrected for age, using the R-package “ppcor” [43].

## Results

**Fig. 1:**
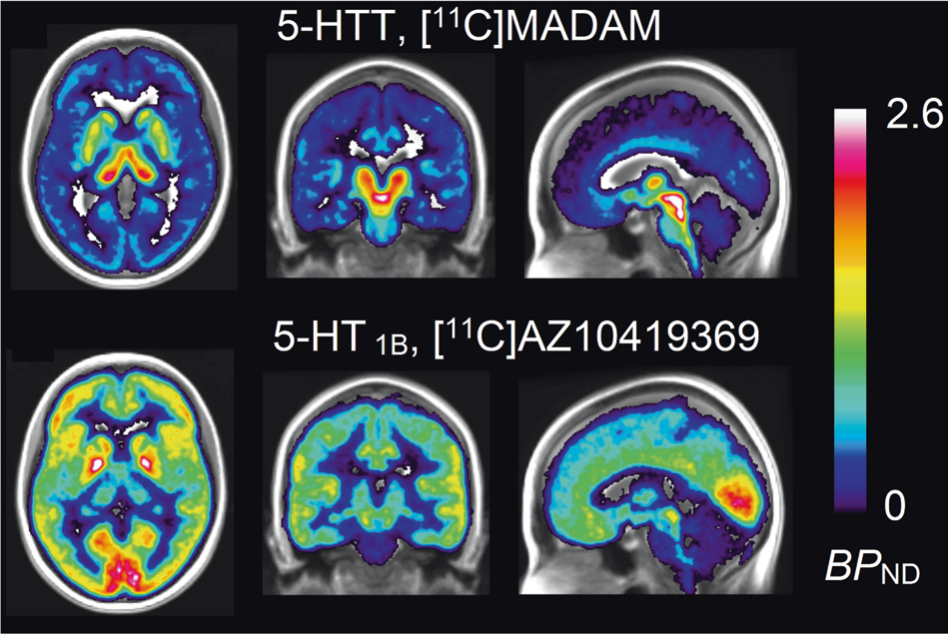
Mean parametric images of [^11^C]MADAM and [^11^C]AZ10419369 examinations overlaid on an averaged MR-image. Images are aligned in the same plane. *BP*_ND_, non-displaceable binding potential.

Average *BP*_ND_ in the examined brain regions is reported for both [^11^C]MADAM and [^11^C]AZ10419369 in Table 1 and visualized in Fig. 1.

**Table 1:** Regional [^11^C]MADAM and [^11^C]AZ10419369 *BP*_ND_ and correlation.

*Region*	[^11^C]MADAM *BP*_ND_ (mean ± SD)	[^11^C]AZ10419369 *BP*_ND_ (mean ± SD)	correlation	*p* value
Temporal cortex	0.26 ± 0.04	0.86 ± 0.10	0.60	0.01
Frontal cortex	0.22 ± 0.04	1.05 ± 0.17	0.64	0.01
Parietal cortex	0.23 ± 0.04	0.95 ± 0.14	0.80	0.0002
Posterior cingulate	0.39 ± 0.07	0.91 ± 0.16	0.44	0.09
Occipital cortex	0.27 ± 0.05	1.21 ± 0.15	0.37	0.16
Anterior cingulate	0.40 ± 0.06	1.04 ± 0.19	0.23	0.40
Amygdala	0.93 ± 0.12	0.93 ± 0.2	0.13	0.62
Caudate Nucleus	0.73 ± 0.12	0.93 ± 0.13	0.29	0.28
Hippocampus	0.38 ± 0.08	0.45 ± 0.12	0.51	0.04
Insula	0.54 ± 0.07	1.05 ± 0.18	0.42	0.11
Putamen	1.12 ± 0.15	1.27 ± 0.18	0.11	0.68
Thalamus	1.17 ± 0.10	0.53 ± 0.09	0.38	0.15
Accumbens	1.04 ± 0.13	1.79 ± 0.3	0.01	0.96
Median raphe	2.88 ± 0.47	0.97 ± 0.33	0.19	0.48
Dorsal raphe	3.15 ± 0.42	1.41 ± 0.31	0.40	0.12

**Fig. 2:**
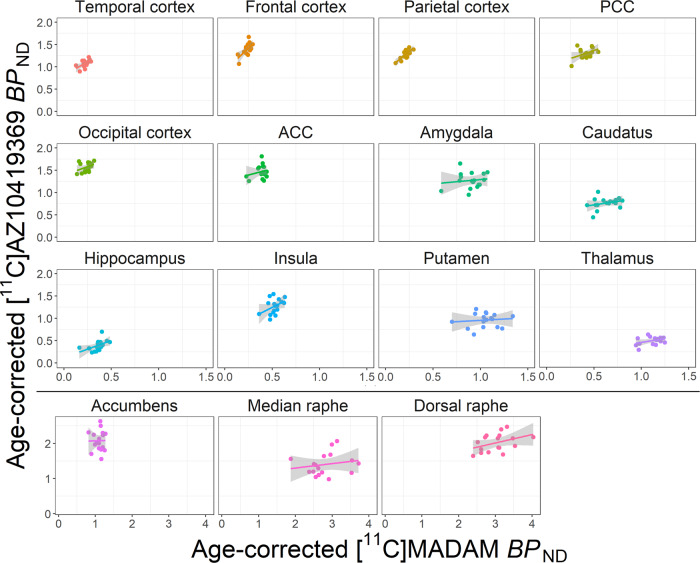
Scatter plots of [^11^C]AZ10419369 and [^11^C]MADAM binding in different brain regions. Plotted BP_ND_ values are corrected for age. For purposes of visualization separate scales have been used in the three lowest panels (Accumbens, median and dorsal raphe nuclei, situated below the black horizontal line). Regression lines with 95% confidence interval in shaded gray. See Supplementary Fig. S3 for a version of the figure with individual scales for each region. ACC Anterior cingulate cortex, PCC Posterior cingulate cortex, BP_ND_, non-displaceable binding potential.

[^11^C]AZ10419369 and [^11^C]MADAM binding was positively correlated within all 15 examined brain regions. In many cortical regions the correlation point estimate was strong, e.g., temporal cortex, r(15) = 0.6, *p* = 0.01 and parietal cortex, r(15) = 0.8, *p* = 0.0002. In most subcortical regions the correlation estimate was negligible, e.g., putamen, r(15) = 0.11, *p* = 0.68 and amygdala, r(15) = 0.13, *p* = 0.62, with hippocampus as the exception, r(15) = 0.51, *p* = 0.04 (Table 1, Fig. 2).

## Discussion

In this PET study, we report on the in vivo association between 5-HTT and 5-HT_1B_ receptor binding in the human brain. We observe a strong positive correlation between the binding estimates in cortical regions and hippocampus, and negligible to weak correlations in subcortical regions, such as the striatum.

In some, but not all, cortical regions 5-HT_1B_ receptor binding correlated with 5-HTT binding. While 5-HTT is only expressed on 5-HT neurons and with a low density in cortex [44], autoradiography studies show a high density of the 5-HT_1B_ receptor in the cerebral cortex [44] where it is expressed both as autoreceptors, and as heteroreceptors on non-serotonergic neurons, including GABAergic and cholinergic neurons [11]. It is not possible to differentiate between auto- and heteroreceptors from PET data, and in rodent neocortex there are studies in support of both 5-HT_1B_ auto- and heteroreceptor availability [11, 45, 46]. Thus, the correlations between cortical 5-HTT and 5-HT_1B_ receptors may entail either the autoreceptor or the heteroreceptor population in cortex, or both. In the hippocampus we observed a correlation of 0.51 between the two examined proteins. Hippocampus is a subcortical brain region with demonstrated 5-HT_1B_ autoreceptor function [11, 47]. It has been suggested that the delay in onset of SSRI antidepressant effect is related to downstream modulation of inhibitory serotonin autoreceptors [48]. This idea is supported by observations of a twofold increase in extracellular serotonin concentration in hippocampus following SSRI exposure in 5-HT_1B_ receptor knockout mice [47], and increased 5-HT_1B_ receptor binding after a single oral dose of SSRI in healthy controls [49]. In line with this reasoning, increased 5-HT_1B_ receptor binding in the hippocampus has been demonstrated with PET after rapid acting MDD treatments such as ketamine [50] and electroconvulsive therapy [51]. In previous PET studies of 5-HT_1B_ receptor binding in unmedicated patients with depression low 5-HT_1B_ receptor binding in hippocampus has been reported [52, 53]. It could be hypothesized that 5-HTT inhibition leads to upregulation of 5-HT_1B_ receptors in projection regions, as has been found in healthy volunteers [49]. Though the relationship between 5-HTT and 5-HT_1B_ receptors in man in vivo needs to be explored further, such dynamics could be leveraged in designing novel treatments schemes.

The discrepancy between the strong correlations observed in cortical regions and the negligible to weak correlations in some subcortical regions could possibly be explained by a lack of autoreceptors in the subcortical regions. Studies reporting on lesioning of serotonergic neurons has shown either increased or not affected 5-HT_1B_ receptor binding in nucleus caudatus and putamen [45, 54, 55], indicating that 5-HT_1B_ receptors in these regions are not autoreceptors. In humans, nucleus caudatus and putamen stands out as the only regions with non-detectable age related decrease in 5-HT_1B_ receptor binding [18]. Thus, we observed correlations in brain regions with documented 5-HT_1B_ autoreceptor expression, but not in regions where the 5-HT_1B_ receptor predominantly is expressed as a heteroreceptor. For the subcortical region thalamus, 5-HT_1B_ receptor localization is relatively unexplored. However, the discrepancy between high thalamic 5-HTT binding and low 5-HT_1B_ receptor binding in this region is well in line with previous reports [44]. Thus, while the 5-HT_1B_ receptor is inhibitory, with decreased serotonin release upon autoreceptor activation, regional differences in 5-HT_1B_ receptor localization may lead to different modulation of neurotransmission in different regions, such as when signaling is stimulated through 5-HT_1B_ receptor inhibition of inhibitory GABA interneurons, as can be seen with 5-HT_1B_ receptor induced increase of dopamine release [56, 57]. Moreover, regionally different changes of 5-HT_1B_ receptor binding have been demonstrated after 5-HTT inhibition, with increased 5-HT_1B_ receptor binding in cortical regions and reduced 5-HT_1B_ receptor binding in raphe nuclei [49].

An important caveat to the discussion above is that [11C]MADAM show very low binding in most cortical regions, with *BP*_ND_s in our data ranging between 0.22 (frontal cortex) and 0.54 (insula). Thus, the signal-to-background is relatively poor, which in theory should make it more difficult to detect a correlation induced by specific binding to the target proteins. Despite this, we observe the strongest correlations in regions with the lowest [11C]MADAM *BP*_ND_ (Table 1). One possible explanation could be a correlation in the radioactive uptake between the two radioligands in the reference region. Such correlation could be caused by similarities in non-specific binding, differing cerebellar anatomy between subjects, or small discrepancies in ROI delineation. If, for instance, an individual has an anatomical variant with wider cerebellar sulci, increasing radioactive spill out to cerebrospinal fluid, this will dilute the uptake for the reference region in both PET experiments. A correlation of radioligand uptake in the reference tissue, regardless of whether it is methodologically induced, or if it originates from similarities in non-specific binding, will have a larger impact on *BP*_ND_ correlation estimates in brain regions with low densities of the target proteins. Supporting this explanation is the observation of a strong correlation between the two radioligands in centrum semiovale, a region purportedly only containing white matter (r = 0.66, *p* = 0.01, controlled for age). An analysis of interindividual variability of radioactive uptake in the non-displaceable compartment would require an arterial input function and is not possible within the present dataset. Such an analysis should be considered as part of the further explorations of the 5-HTT and 5-HT_1B_ receptors in vivo.

The age range of the study participants was relatively wide (23-75 years). The binding estimates for both radioligands is known to be inversely correlated with age [18, 42]. In the statistical analysis we have attempted to correct for this using age as a covariate. The age effect may in part be caused by spill-out of radioactivity due to age related decrease in gray matter volume. In order to further explore this, we performed an analysis correcting for the volume of each brain region instead of age. In this analysis the correlation estimates were attenuated in cortical regions and strengthened in thalamus (Supplementary Table S1).

Twelve of the seventeen subjects performed both the [11C]MADAM and [^11^C]AZ10419369 examinations during the same day. For five subjects, logistical difficulties made same day experiments impossible. Three subjects therefore performed the examinations with one day apart and two subjects with seven days apart. An analysis restricted to the 12 subjects with same day experiments showed similar correlation estimates compared with the full sample in most examined brain regions (Supplementary Table S2).

In conclusion, we found moderate to strong correlations between 5-HTT and 5-HT_1B_ receptors in brain regions with documented distributions of 5-HT_1B_ autoreceptors. Such associations are in line with previously described functional relationship between two key regulators of the serotonin system. However, the fact that the strongest correlations were found in regions with the lowest signal-to-background ratio raises the caveat that associations might be, in part, induced by factors not related to specific binding of the radioligands. The clinical potential of the interplay between 5-HTT and 5-HT_1B_ receptors should be investigated in patients treated with SSRI.

## Supplementary information

Supplemental material
