# Supplementary material for: In vivo correlation of serotonin transporter and 1B receptor availability in the human brain: a PET study
Source: Neuropsychopharmacology. 2022 Jul 11;47(10):1863–8. doi: 10.1038/s41386-022-01369-3 (PMC9372190; doi:10.1038/s41386-022-01369-3)
Supplement: Supplementary file 1 — Supplemental material [file 41386_2022_1369_MOESM1_ESM.pdf]

In vivo correlation of serotonin transporter and 1B receptor availability in the human brain: a PET study

Supplemental Material

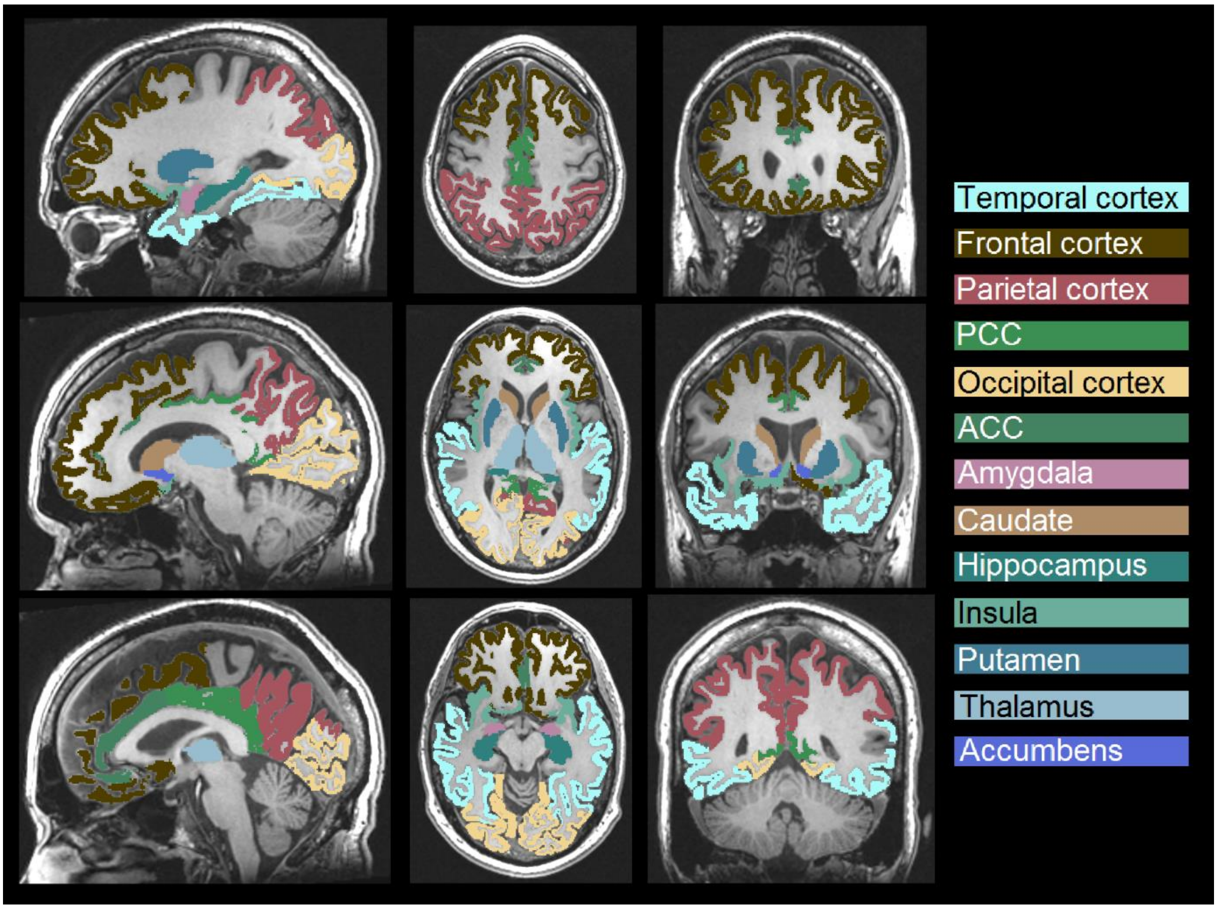

Supplemental figure S1. MRI for one subject with regions of interest overlaid. From top to bottom, the sagittal images (left column) move from a lateral to a medial view, the axial images (middle column) from a superior to inferior view, the coronal images (right column) from an anterior to a posterior view. ACC, anterior cingulate cortex; PCC, posterior cingulate cortex

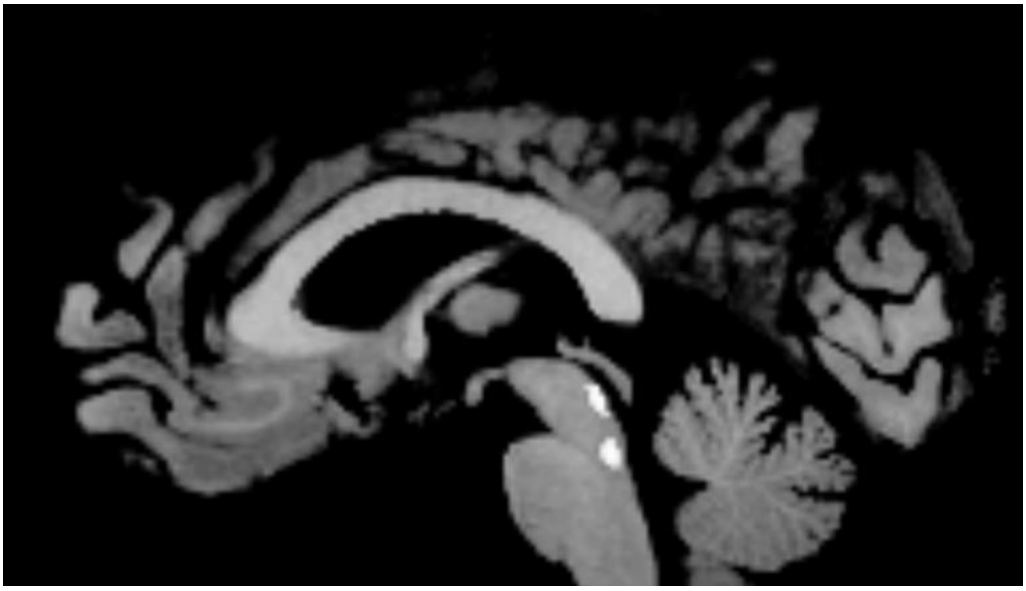

Supplemental figure S2. Sagital view of MRI for one subject with the median and dorsal raphe masks overlaid in white. Both masks situated in the brainstem, dorsal raphe mask directly anterior of the cerebral aqueduct; median raphe mask below

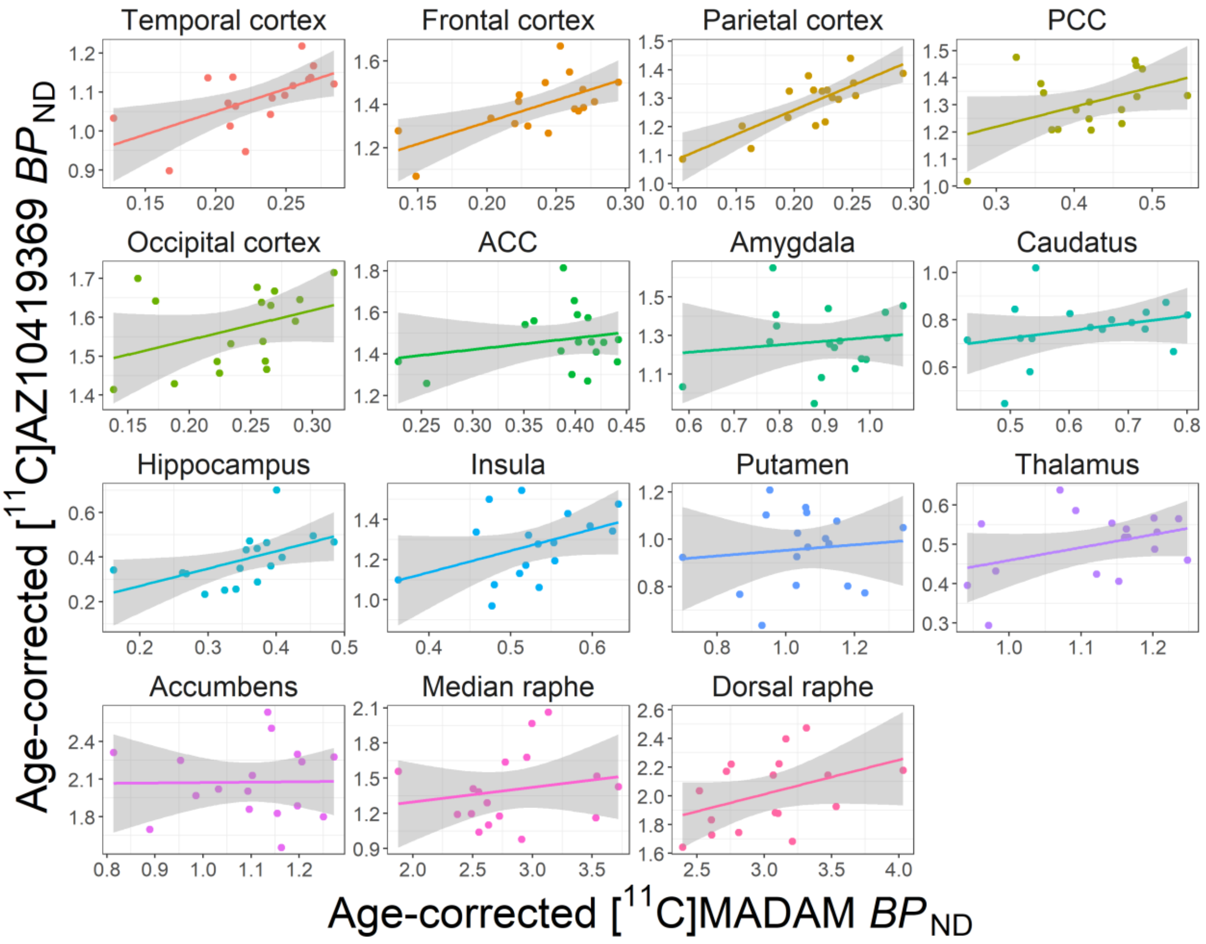

Supplemental figure S3. Scatter plots of [^{11}C]AZ10419369 and [^{11}C]MADAM binding in different brain regions. Plotted BPND values are corrected for age. Regression line with 95% confidence interval in shaded gray. ACC, Anterior cingulate cortex; PCC, Posterior cingulate cortex; BP_{ND}, non-displaceable binding potential.

Table S1. [^{11}C]MADAM and [^{11}C]AZ10419369 BP_{ND} and volume corrected correlations

Region	[^{11}C]MADAM BP_{ND} (mean±SD)	[^{11}C]AZ10419369 BP_{ND} (mean±SD)	correlation	p-value
Temporal cortex	0.26±0.04	0.86±0.10	0.33	0.21
Frontal cortex	0.22±0.04	1.05±0.17	0.61	0.01
Parietal cortex	0.23±0.04	0.95±0.14	0.47	0.06
Posterior cingulate	0.39±0.07	0.91±0.16	0.39	0.13
Occipital cortex	0.27±0.05	1.21±0.15	0.11	0.69
Anterior cingulate	0.40±0.06	1.04±0.19	0.16	0.54
Amygdala	0.93±0.12	0.93±0.2	0.17	0.53
Caudate Nucleus	0.73±0.12	0.93±0.13	0.22	0.42
Hippocampus	0.38±0.08	0.45±0.12	0.54	0.03
Insula	0.54±0.07	1.05±0.18	0.24	0.37
Putamen	1.12±0.15	1.27±0.18	0.18	0.5
Thalamus	1.17±0.10	0.53±0.09	0.52	0.04
Accumbens	1.04±0.13	1.79±0.3	0.04	0.87

Table S2. [^{11}C]MADAM and [^{11}C]AZ10419369 BP_{ND} and cor, only same day PET (n=12)

Region	[^{11}C]MADAM BP_{ND} (mean±SD)	[^{11}C]AZ10419369 BP_{ND} (mean±SD)	correlation	p-value
Temporal cortex	0.26±0.04	0.85±0.11	0.6	0.05
Frontal cortex	0.22±0.05	1.05±0.18	0.68	0.02
Parietal cortex	0.23±0.04	0.94±0.14	0.76	0.01
Posterior cingulate	0.37±0.07	0.9±0.15	0.33	0.33
Occipital cortex	0.26±0.05	1.21±0.13	0.17	0.62
Anterior cingulate	0.4±0.07	1.02±0.21	0.27	0.41
Amygdala	0.92±0.13	0.91±0.22	0.13	0.71
Caudate Nucleus	0.73±0.12	0.9±0.14	0.24	0.48
Hippocampus	0.37±0.08	0.44±0.13	0.48	0.13
Insula	0.53±0.07	1±0.19	0.51	0.11
Putamen	1.1±0.15	1.24±0.18	0.07	0.84
Thalamus	1.16±0.11	0.51±0.1	0.24	0.47
Accumbens	1.05±0.14	1.79±0.36	0.03	0.93
Median raphe	2.82±0.51	1±0.29	0.29	0.39
Dorsal raphe	3.18±0.5	1.41±0.32	0.44	0.17
